# A Global Perspective on Metabolic Dysfunction-Associated Steatotic Liver Disease: From Molecular Mechanisms to Therapeutic Strategy Innovation

**DOI:** 10.3390/nu18040679

**Published:** 2026-02-19

**Authors:** Yanhao Qiu, Juan Carlos Laguna, Marta Alegret, Laia Vilà

**Affiliations:** 1 Department of Pharmacology, Toxicology and Therapeutic Chemistry, School of Pharmacy and Food Science, University of Barcelona, 08028 Barcelona, Spain; yqiuqiux231@alumnes.ub.edu (Y.Q.); jclagunae@ub.edu (J.C.L.); alegret@ub.edu (M.A.); 2 Spanish Biomedical Research Centre in Physiopathology of Obesity and Nutrition (CIBEROBN), Instituto de Salud Carlos III, 28029 Madrid, Spain; 3 Institute of Biomedicine, University of Barcelona, 08028 Barcelona, Spain

**Keywords:** MASLD, pathogenesis, sexual dimorphism, animal models, therapeutics

## Abstract

Metabolic Dysfunction-Associated Steatotic Liver Disease (MASLD) is a prevalent global health issue driven by metabolic syndrome, with cardiovascular disease being the leading cause of mortality. This review synthesizes current knowledge on its multifactorial pathogenesis, the impact of sexual dimorphism, and key experimental models. The progression of MASLD involves interconnected pathways including dysregulated de novo lipogenesis, insulin resistance, mitochondrial dysfunction, gut dysbiosis, ferroptosis, and genetic and epigenetic predispositions. These mechanisms not only promote hepatic injury but also accelerate atherosclerosis. Notably, MASLD exhibits significant sexual dimorphism, influenced by physiological differences, sex hormones, genetic factors, and the microbiome. The study of these complex processes relies mostly on dietary-induced animal models, particularly in rats, which effectively recapitulate features of the human disease. Given the multifaceted nature of MASLD, the therapeutic focus is shifting from monotherapies to combination or dual-target strategies. To enable this transition, refinement of preclinical models is essential to better understand and target this complex disorder.

## 1. Introduction

Liver abnormalities, including hepatic steatosis, steatohepatitis, fibrosis, and hepatocellular carcinoma, represent a significant health concern, with ectopic fat deposition in the liver being a major research focus [1]. Hepatic steatosis was previously classified into alcoholic (AFLD) and non-alcoholic fatty liver disease (NAFLD), but recently, NAFLD has been redefined as metabolic dysfunction-associated steatotic liver disease (MASLD), encompassing individuals with hepatic steatosis who have at least one cardiometabolic risk factor (Table 1) and consume low amounts of alcohol. This new classification highlights the metabolic origins of the disease and distinguishes these patients from those who consume moderate to high amounts of alcohol [2]. MASLD affects approximately 30% of the global population, particularly individuals with obesity and type 2 diabetes [3,4,5]. MASLD progresses from metabolic dysfunction-associated steatotic liver (MASL), marked by the excess of neutral lipid (triglyceride and cholesteryl esters) accumulation, to metabolic dysfunction-associated steatohepatitis (MASH), which involves inflammation, cell death, and increased risk of fibrosis and end-stage liver disease, including cirrhosis and liver cancer [6]. Nevertheless, cardiovascular disease (CVD) remains the leading cause of mortality in MASLD patients [7].

Despite its prevalence, MASLD is underdiagnosed and undertreated due to limited diagnostic methods. Liver enzymes, mainly alanine aminotransferase (ALT) and aspartate aminotransferase (AST), are non-invasive and inexpensive biomarkers commonly used for MASLD screening, although elevated levels of these enzymes are not sufficient to confirm the pathology [8]. Recent guidelines suggest using a simple fibrosis score, such as the Fibrosis 4 Index [FIB-4], as an initial screening tool for advanced liver fibrosis [9,10]. Research has shown that FIB-4 has a high negative predictive value in excluding advanced liver fibrosis, but its positive predictive value is relatively low. FIB-4’s diagnostic performance is suboptimal in patients over 65 and those with type 2 diabetes mellitus, where reduced sensitivity may lead to missed diagnoses [11]. In these cases, liver stiffness measurement (LSM) by vibration-controlled transient elastography, a non-invasive imaging technique that quantifies liver fibrosis by measuring tissue stiffness, is recommended as a complementary second-line test. Similarly, patients with elevated FIB-4 scores are advised to undergo LSM, for more accurate risk stratification [12]. Nevertheless, the diagnostic gold standard for MASLD remains histological evaluation through liver biopsy.

Regarding drug therapy, the FDA granted accelerated approval to resmetirom (an oral liver-targeted thyroid hormone receptor-beta agonist) in March 2024 for the treatment of adults with MASH and moderate to advanced fibrosis (F2–F3) [13,14]. Semaglutide, an injectable glucagon-like peptide-1 receptor agonist, received FDA accelerated approval later in 2025 for noncirrhotic MASH with moderate to advanced liver fibrosis, making it the second drug approved for this indication after resmetirom [15]. Both approvals are based on histologic endpoints in phase 3 clinical trials such as resolution of steatohepatitis without fibrosis worsening and improvement in fibrosis with no worsening of steatohepatitis, but they differ mechanistically: resmetirom targets liver metabolism, while semaglutide primarily induces weight loss and glycemic control [15,16]. The regulatory pathways for both drugs involved accelerated approval due to unmet medical need and reliance on surrogate endpoints reasonably likely to predict clinical benefit. In clinical trials, resmetirom showed significant improvements in NASH resolution and fibrosis reduction in about 25–30% of treated patients, indicating that not all patients respond adequately [17]. There is limited evidence supporting its use outside the fibrosis window F2-F3, and patients with cirrhosis (F4) were excluded from trials, so benefits in those populations remain unproven. Moreover, there is currently no drug approved for the treatment of isolated steatosis, with options limited to the implementation of healthy lifestyle, including dietary measures.

Dual-targeted therapies are particularly valuable in addressing the limitations of single-targeted treatments such as resmetirom by simultaneously acting on multiple pathological processes. This is especially relevant in the context of metabolism-related diseases, including MASLD, in which pathological factors like insulin resistance (IR), oxidative stress, and inflammation often coexist and interact, exacerbating disease progression [16,18,19]. Dual-targeted therapies can comprehensively intervene in these processes by modulating multiple pathways including bile acid synthesis, de novo lipogenesis (DNL), insulin resistance, lipid oxidation and inflammation, all of them involved in MASLD development and progression. Preclinical models play a crucial role in the development of new therapeutic strategies for MASLD. Identifying an animal model that accurately replicates the pathological alterations observed in humans at various disease stages is essential. In this sense, rat dietary models better recapitulate the origin of the human disease and understanding the dietary components and their effects on histological outcomes and metabolic disturbances is fundamental for both basic and translational research.

Finally, it has to be considered that sex differences have a significant impact on disease onset, progression, clinical manifestations and treatment outcomes. Men are more likely to develop MASLD, and show higher levels of steatosis and fibrosis, while in women, mainly oestrogen levels and differential fat distribution may protect against this pathology by regulating metabolic, inflammatory and oxidative pathways [20,21,22]. Specially, oestrogen levels are associated with a reduction in DNL and an increase in lipid oxidation, both of which prevent lipid deposition in liver. Meanwhile, fat distribution influences insulin sensitivity and inflammation, further modulating disease risk. In addition, sex differences in atherosclerosis and CVD are also significant [23,24]. These differences suggest that sex-specific treatment and prevention strategies for MASLD are critical.

**Figure 1:**
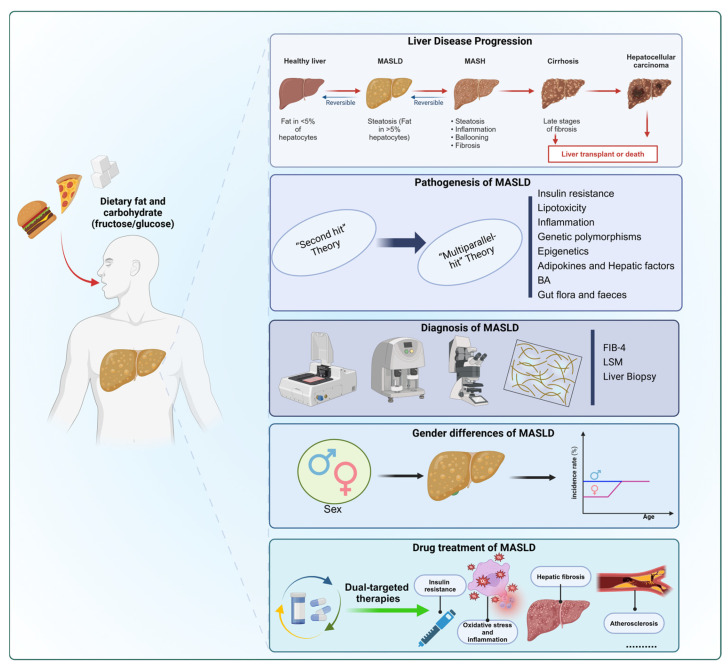
A Systems Biology View of MASLD. The figure illustrates the MASLD spectrum from simple steatosis to hepatocellular carcinoma and the metabolic pathways involved in its development and progression. It highlights the importance of early diagnosis, primarily through non-invasive clinical and biochemical markers, including the fibrosis-4 index (FIB-4) and liver stiffness measurement (LSM), which are key tools for disease assessment and risk stratification. The figure also emphasizes sexual dimorphism in MASLD, with men being more likely to develop the disease and to exhibit higher levels of steatosis and fibrosis. Finally, given the complexity and multifactorial nature of MASLD, the figure illustrates the rationale for the development of dual-targeted therapies that simultaneously act on multiple pathogenic processes. Abbreviations: BA, Bile acids; FIB-4, Fibrosis 4 index; LSM, liver stiffness measurement; MASH, metabolic dysfunction-associated steatohepatitis; MASLD, metabolic dysfunction-associated steatotic liver disease. This image was created with BioRender (https://biorender.com/).

Taken together, this review provides a comprehensive overview of MASLD pathogenesis, highlights sex-specific differences in disease progression, and discusses emerging pharmacological treatments, with particular focus on dietary liver disease rat models that serve as indispensable tools for preclinical drug testing (Figure 1).

## 2. Methods

Relevant literature was identified through searches of the PubMed database, covering publications from inception to 2025 and restricted to articles published in English. Searches were performed using a combination of Medical Subject Headings terms and free-text keywords related to MASLD, including disease definition, metabolic abnormalities, pathogenic mechanisms, experimental models, and therapeutic approaches. To complement keyword-based retrieval and reduce the risk of missing influential studies, Connected Papers (https://www.connectedpapers.com/https://www.connectedpapers.com/; accessed from 10 March 2025 to 5 February 2026) was used to build citation-based literature networks and to identify conceptually related and highly relevant publications. In addition, the literature indexed in PubMed was analyzed using the citexs platform (https://www.citexs.com/; accessed from 10 March 2025 to 5 February 2026), with particular emphasis on studies published within the past five years, in order to characterize the evolution of the field and identify shifts in research focus and emerging hotspots. Original research articles and review papers were included, and additional relevant studies were retrieved by screening reference lists. Study selection was performed by the first author, with all authors involved in the critical evaluation and revision of the manuscript.

## 3. MASLD Overview

### 3.1. Definition of MASLD

MASLD encompasses a broad spectrum of liver conditions that ranges from hepatic steatosis to steatohepatitis, liver fibrosis or even liver cancer. These hepatic alterations are strongly associated with cardiometabolic risk factors, such as obesity, type 2 diabetes, and dyslipidaemia. MASLD, which is considered a major health concern, is closely related to dysfunctional lipid metabolism, oxidative stress, IR and inflammation [25]. With the emergence of new findings, the traditional “second hit” theory (proposing that initial hepatic fat accumulation due to metabolic dysfunction is followed by a second hit such as oxidative stress) is no longer sufficient to explain the complex pathogenesis of MASLD. Thus, a new concept of “multi-parallel hit” has been developed, involving IR, lipotoxicity, inflammation, genetic polymorphisms and epigenetics, adipokines, hepatic factors, bile acid (BA) and gut microbiota acting simultaneously rather than sequentially [26,27,28,29].

Aligned with the multiple-hit hypothesis, in 2020, an international panel of experts proposed renaming NAFLD to MAFLD. This proposal was based on recognizing the pathogenic role of metabolic dysfunction, the multifactorial nature of disease progression, and its associated systemic extrahepatic complications [30]. Building on this concept, three major international liver societies subsequently recommended a unified nomenclature in 2023, introducing steatotic liver disease (SLD) as an umbrella term encompassing liver diseases characterized by hepatic fat accumulation as a shared mechanism of injury, and renaming MAFLD as MASLD [2]. Within the SLD framework, patients are further subclassified according to the presence of metabolic dysfunction and the extent of alcohol consumption. Specifically, SLD comprises: (1) MASLD, which replaces NAFLD and includes individuals with hepatic steatosis and at least one cardiometabolic risk factor (Table 1) who consume less than two standard drinks per day for females and three for males; (2) alcohol-related liver disease (ALD), defined by alcohol intake exceeding five drinks per day for females and six for males; (3) metabolic dysfunction-associated alcohol-related liver disease (MetALD), referring to patients with at least one metabolic risk factor and moderate alcohol consumption ranging from two to five drinks per day for females and three to six for males; and (4) other causes of steatosis, such as specific drug-induced liver injury [31].

The introduction of MetALD represents an important refinement of disease classification, delineating a clinically relevant population with hepatic steatosis who fulfil the diagnostic criteria for MASLD but consume alcohol beyond the permitted threshold for MASLD, while remaining below that required for a diagnosis of classical ALD. This category reflects the frequent coexistence of metabolic dysfunction and alcohol exposure and recognizes their synergistic effects in promoting liver injury, fibrosis progression, and adverse clinical outcomes.

Emerging epidemiological evidence indicates a high concordance between the definitions of NAFLD and MASLD, with approximately 99% of NAFLD patients meeting the MASLD criteria. However, patients classified under MASLD exhibit a slightly higher mortality risk, attributed to the presence of cardiometabolic risk factors [32,33]. The updated SLD nomenclature, together with the formal recognition of MetALD, enhances diagnostic precision, refines risk stratification, and provides a more robust framework for clinical management and translational research.

**Table 1:** MASLD standards of judgement based on the 2023 Delphi Consensus [34].

	**Cardiometabolic Risk Factors**	**Standard of Judgement**
①	Body mass index	BMI ≥ 25 kg/m^2^ (≥23 kg/m^2^ in Asians) or waist circumference: Men > 94 cm, Women > 80 cm (or ethnicity-adjusted)
②	Glucose-Related	Fasting serum glucose ≥ 100 mg/dL (≥5.6 mmol/L) or 2 h post-load glucose level ≥ 140 mg/dL (≥7.8 mmol/L) or HbA1c ≥ 5.7% or on specific drug treatment
③	Blood Pressure	Blood pressure ≥ 130/85 mmHg or on specific drug treatment
④	Triglycerides	Plasma triglycerides ≥ 150 mg/dL (≥1.70 mmol/L) or on specific drug treatment
⑤	HDL Cholesterol	For men: HDL < 40 mg/dL (<1.0 mmol/L); for women: HDL < 50 mg/dL (<1.3 mmol/L) or on specific drug treatment

BMI, body mass index; HbA1c, glycated hemoglobin A1c; HDL, high-density lipoprotein; MASLD, metabolic dysfunction-associated steatotic liver disease.

### 3.2. Pathogenesis and Progression of MASLD

**Figure 2:**
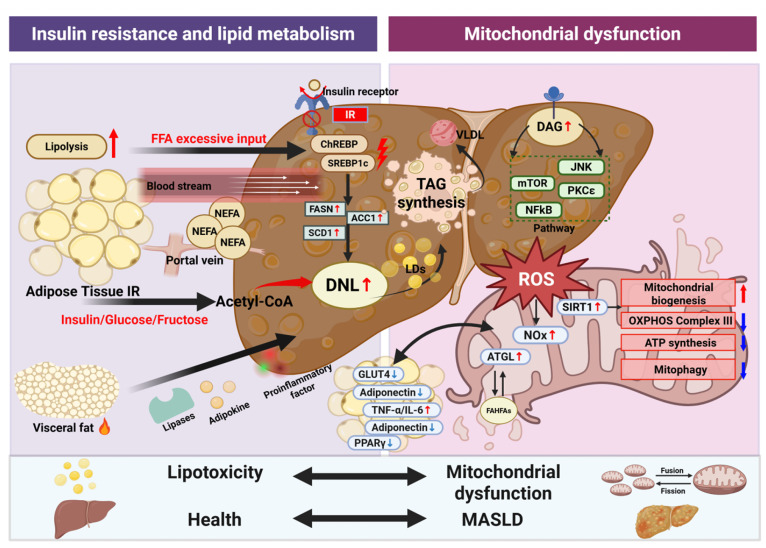
Mechanisms linking lipid metabolism disorders and mitochondrial dysfunction to MASLD. The core driver of hepatic steatosis is the dysregulation of hepatic lipid homeostasis, characterized by exacerbated DNL and increased NEFA flux from adipose tissue. Systemic and hepatic IR acts as a pivotal link: in adipose tissue, IR causes uncontrolled lipolysis and NEFA release; in the liver, selective IR paradoxically enhances DNL via transcription factors SREBP-1c and ChREBP. Furthermore, mitochondrial dysfunction induced by lipid overload leads to excessive reactive ROS production, promoting oxidative stress, hepatocyte injury, and inflammation. These three pathways form a self-amplifying vicious cycle that propels MASLD progression. Abbreviations: ACC, acetyl-CoA carboxylase; ATGL, Adipose triglyceride lipase; ATP, adenosine triphosphate; ChREBP, carbohydrate response element binding protein; DAG, diacylglycerol; DNL, de novo lipogenesis; FASN, fatty acid synthase; GLUT4, glucose transporter 4; IL-6, interleukin-6; IR, insulin resistance; JNK, *c*-Jun N-terminal kinase; LD, lipid droplet; MASLD, metabolic dysfunction-associated steatotic liver disease; mTOR, mammalian target of rapamycin; NEFA, non-esterified fatty acid; NF-Κb, nuclear factor kappa-B; Nox, NADPH oxidase; OXPHOS, oxidative phosphorylation; PKCε, protein kinase Cε; PPARγ, peroxisome proliferator-activated receptor gamma; SCD-1, stearoyl-CoA desaturase 1; SIRT1,sirtuin 1; SREBP-1c, sterol regulatory element-binding protein 1c; TAG, triacylglycerol; TNFα, tumour necrosis factor α; VLDL, very low-density lipoproteins. This image was created with BioRender (https://biorender.com/).

**Figure 3:**
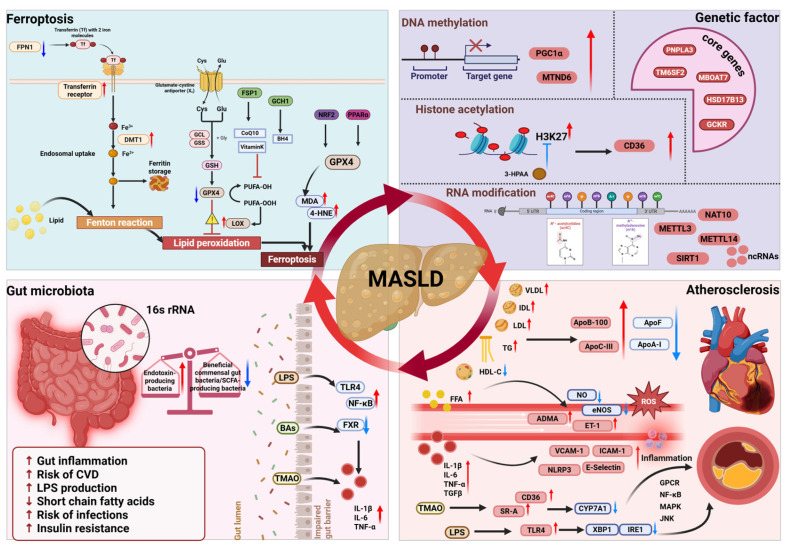
Multi-Mechanistic Crosstalk in MASLD: A Network Linking Ferroptosis, Epigenetics, Gut Dysbiosis and Atherosclerotic Pathogenesis. The interplay between gut dysbiosis, hepatic ferroptosis, and genetic/epigenetic susceptibility creates a self-amplifying loop within the liver, exacerbating lipid accumulation, oxidative stress, and inflammation. This hepatic metabolic derangement breaks systemic homeostasis, exporting pro-atherogenic signals, including dyslipidaemia, endothelial dysfunction, and microbial metabolites like TMAO that directly fuel the development and progression of atherosclerosis, thereby closing a detrimental liver-vascular axis. Abbreviations: ADMA, asymmetric dimethylarginine; BH4, tetrahydrobiopterin4; CoQ10, coenzyme Q10; CYP7A1, cholesterol 7 alpha-hydroxylase; DMT1, divalent metal transporter 1; ET-1, endothelin-1; FPN1, ferroportin 1; FSP1, ferroptosis suppressor protein 1; GCH1, GTP cyclic hydrolase 1; GCKR, glucokinase regulator; GPX4, glutathione peroxidase 4; GSH, glutathione; 4-HNE, 4-hydroxynonenal; HDL; high-density lipoprotein; HSD17B13, hydroxysteroid 17-beta dehydrogenase 13; ICAM-1, intercellular adhesion molecule-1; IDL, intermediate-density lipoprotein; LDL, low-density lipoprotein LOX, lipoxygenase; LPS, lipopolysaccharide; MASLD, metabolic dysfunction-associated steatotic liver disease; MDA, malondialdehyde; MBOAT7, membrane-bound O-acyltransferase domain-containing 7; METTL3, methyltransferase-like 3; METTL14, methyltransferase-like 14; MT-ND6, mitochondrially encoded NADH dehydrogenase 6; NAT10, *N*-acetyltransferase 10; Nrf2, nuclear factor erythroid 2-related factor 2; PGC1α, peroxisome proliferator activated receptor coactivator 1 alpha; PNPLA3, patatin-like phospholipase domain containing protein 3; PPARα, peroxisome proliferator activated receptor alpha; PUFA, polyunsaturated fatty acids; TG, triglyceride; TLRs, toll like receptors; TMAO, trimethylamine *N*-oxide; TM6SF2, transmembrane 6 superfamily member 2; SR-A, scavenger receptor-A; TFR1, transferrin receptor 1; VCAM-1, vascular cell adhesion molecule-1; VLDL, very low-density lipoprotein. This image was created with BioRender (https://biorender.com/).

The pathophysiology of MASLD is complex and multifactorial. Across all stages of the disease, the initial and central event is excessive hepatic lipid accumulation, primarily driven by increased de novo lipogenesis (DNL) and enhanced hepatic uptake and storage of non-esterified fatty acids (NEFAs) released through adipose tissue lipolysis. Additional etiopathogenetic mechanisms include mitochondrial dysfunction and oxidative stress, dysregulation of the gut–liver axis and intestinal microbiota, ferroptosis, and genetic and epigenetic factors that modulate disease susceptibility and progression [35] (Figure 2 and Figure 3). Each of these mechanisms is described in detail in the following sections.

#### 3.2.1. The Core Effect of De Novo Lipogenesis in MASLD

Under physiological conditions, hepatic lipid pools are tightly regulated and maintained in a dynamic equilibrium. Approximately 60% of hepatic lipids are derived from NEFAs released via lipolysis by peripheral adipose tissue, 15% from dietary fat uptake, 15% from the liver uptake of remnant lipoproteins and the remaining 10% through hepatic DNL [36]. In MASLD, this equilibrium is profoundly disrupted, and excessive NEFA delivery from adipose tissue combined with pathologically elevated hepatic DNL collectively drives hepatic steatosis [37].

DNL is an anabolic process that converts simple carbohydrates (such as glucose and fructose) and other metabolic intermediates (including lactate, acetate, and amino acids) into fatty acids [38]. DNL is initiated when excess substrate availability, relative to cellular energy demands, leads to increases in mitochondrial citrate, which is exported from mitochondria into the cytosol. This cytosolic citrate is then converted into fatty acids by a series of biosynthetic reactions catalyzed by ATP-citrate lyase (ACLY), acetyl-CoA carboxylase (ACC; also known as ACACA) and fatty acid synthase (FAS; also known as FASN) [37]. Fructose is a key substrate for DNL, abundant in typical Western diets, which bypasses the primary regulatory step of glycolysis and is rapidly phosphorylated by ketohexokinase (KHK), thereby increasing the net production of citrate in liver cells and promoting DNL [39]. Furthermore, high dietary fructose intake activates the transcription factors sterol regulatory element-binding protein 1c (SREBP-1c) and carbohydrate response element-binding protein (ChREBP), leading to the upregulation of key DNL enzymes, driving excessive triglyceride (TG) synthesis and deposition in the liver [40].

In patients with MASLD, hepatic DNL is markedly increased and exhibits an inverse correlation with hepatic insulin sensitivity. In these patients, DNL may contribute to as much as 40% of hepatic lipid accumulation under postprandial conditions [41].

#### 3.2.2. IR: The Pivotal Link Connecting Adipose Tissue to Hyperactive Hepatic DNL

IR plays a central role in the pathogenesis and progression of MASLD. It connects peripheral adipose tissue dysfunction with hepatic metabolic dysregulation through a phenomenon termed “selective IR,” thereby establishing a self-amplifying vicious cycle [42].

Adipose tissue is a primary site of IR development, particularly in visceral depots. Under physiological conditions, insulin potently suppresses lipolysis. However, in states of IR, adipocytes become resistant to this anti-lipolytic effect, leading to uncontrolled hydrolysis of stored TG and a consequent excessive release of NEFAs into the portal circulation [43]. These NEFAs are directly delivered to the liver, where they serve as the principal substrate for hepatic TG resynthesis. Although in IR states there is an increase in the secretion of very low-density lipoproteins (VLDL), it is insufficient to compensate for the enhanced TG synthesis and fatty acid influx, resulting in TG retention within hepatocytes and further promoting steatosis. Clinical evidence indicates that this defective inhibition of lipolysis is closely correlated with the severity of hepatic steatosis and the risk of advanced fibrosis [44].

Hepatic IR is selective, meaning that it is pathway-specific. Thus, while the liver becomes resistant to insulin-mediated suppression of gluconeogenesis, contributing to fasting hyperglycaemia, the lipogenic actions of insulin remain active or are even enhanced under hyperinsulinemic conditions. This sustained insulin signalling activates SREBP-1c and ChREBP, [45] leading to a marked upregulation of lipogenic enzymes such as ACC, FASN, and stearoyl-CoA desaturase 1 (SCD-1) [46,47]. Notably, ACC expression is reported to be increased over 8-fold in the livers of MASLD patients compared to individuals with normal liver function [46,48].

Furthermore, hepatic lipid accumulation promotes the formation of toxic lipid intermediates, most notably diacylglycerol (DAG) and ceramides. The accumulation of DAG activates protein kinase Cε (PKCε), which in turn phosphorylates and inhibits the insulin receptor, further impairing hepatic insulin signalling and exacerbating systemic IR [49,50]. Lipid intermediates also activate other signalling cascades in the liver, such as those involving mammalian target of rapamycin (mTOR) and *c*-Jun *N*-terminal kinase (JNK), which are closely associated with MASLD progression [51,52]. Following the metabolic dysregulation induced by IR, adipose tissue dysfunction further aggravates the scenario through altered secretion of adipokines and proinflammatory cytokines (e.g., reduced adiponectin, elevated TNF-α and IL-6), which exacerbates systemic and hepatic inflammation [53,54,55]. At the cellular level, these processes converge to promote oxidative stress, endoplasmic reticulum stress, and mitochondrial dysfunction, creating a lipotoxic environment that drives hepatocyte injury, apoptosis, and ultimately the progression to MASH and fibrosis [56,57].

#### 3.2.3. Mitochondrial Dysfunction and Oxidative Stress in MASLD Pathogenesis

Mitochondrial dysfunction has been established as a pivotal mechanism driving the progression of MASLD, contributing significantly to hepatic lipid accumulation, inflammation, and fibrosis. As the primary sites for fatty acid β-oxidation and oxidative phosphorylation (OXPHOS), mitochondria are central to cellular energy metabolism. Their functional integrity is crucial, and impairment is now recognized as a critical factor in the transition from simple steatosis to MASH [58,59]. In the early stages of MASLD, mitochondria undergo a phase of compensatory adaptation: in response to the overload of NEFAs, they upregulate β-oxidation and TCA cycle activity in an attempt to manage the lipid surplus. However, persistent lipid influx eventually overwhelms mitochondrial metabolic capacity, leading to a decline in oxidative function in advanced MASH [39].

As a direct consequence of mitochondrial overload, electron transport chain efficiency is compromised, particularly at complexes I and III, resulting in increased electron leakage and elevated reactive oxygen species (ROS) production [60,61]. The resulting oxidative stress not only directly damages mitochondrial components, including DNA, proteins, and membranes, leading to ultrastructural abnormalities such as swelling and cristae disruption, but also perpetuates a cycle of further ROS generation [62]. The pathological impact of ROS extends beyond the mitochondria. Oxidative stress activates stress-sensitive signalling pathways, such as JNK and nuclear factor kappa beta (NF-κB), and promotes lipid peroxidation, culminating in hepatocyte apoptosis via mechanisms including cytochrome c release, a key lipotoxic event [63].

Furthermore, mitochondrial quality control mechanisms are frequently impaired in MASH. Mitophagy and mitochondrial biogenesis are disrupted, partly due to the inflammatory suppression of AMP-activated protein kinase (AMPK), a central regulator of energy homeostasis [64,65]. For instance, TNF-α has been shown to suppress AMPK activity, which not only disrupts mitochondrial turnover but also removes its inhibitory phosphorylation of ACC, thereby paradoxically accelerating DNL and disease progression [66,67].

In conclusion, the interplay between mitochondrial dysfunction and oxidative stress creates a self-reinforcing cycle that propels MASLD pathogenesis. These findings underscore that those therapeutic strategies aimed at improving mitochondrial function and restoring redox balance may hold significant promise for mitigating disease progression.

#### 3.2.4. Ferroptosis

Oxidative stress is a key driver of ferroptosis, a recently discovered iron-dependent form of regulated cell death driven by lipid peroxidation, involved in the pathogenesis of MASLD [68]. Ferroptosis requires iron overload in cells, usually as a consequence of increased iron absorption due to dysregulation of transferrin and transferrin receptor 1 (TFR1) and reduced iron storage.

The elevated labile iron pool (Fe^2^) subsequently initiates Fenton reactions, generating hydroxyl radicals (·OH) that oxidize PUFA-containing membrane phospholipids. This oxidative cascade is further amplified by lipoxygenase (LOX)-mediated peroxidation of PUFA, yielding cytotoxic lipid peroxidation products (LPO) that compromise membrane integrity [69].

The glutathione peroxidase 4 (GPX4)-glutathione (GSH) axis constitutes the primary defence against oxidative stress and ferroptosis. GPX4 catalyzes the reduction in lipid hydroperoxides to nontoxic alcohols, thereby interfering with the lipid peroxidation chain reaction [70,71,72]. In this sense, direct GPX4 inactivation triggers ROS production and ferroptosis, marked by accumulation of peroxidation byproducts including malondialdehyde (MDA) and 4-hydroxynonenal (4-HNE). Recently, it has been demonstrated that MASLD progression is accompanied by profound disturbances in these regulatory mechanisms, most notably a reduction in GPX4 levels, in both humans and animal models [73,74,75] (Figure 3). Therefore, strategies aimed at reducing ferroptosis may help to slow the progression of MASLD. In this sense, treatment of *ob*/*ob* mice with ferrostatin-1 (a ferroptosis inhibitor) decreases MDA levels and restores GPX4 activity, mitigating iron overload and, thereby reducing liver inflammation and fibrosis [76].

Interestingly, PPARα activation also demonstrates hepatoprotective effects by attenuating ferroptosis through its binding to the Gpx4 promoter, suggesting direct transcriptional regulation of this key ferroptosis suppressor [77]. Globally, these results suggest that ferroptosis inhibition may represent a promising therapeutic strategy. However, given the intricate interplay between iron metabolism, lipid peroxidation, and antioxidant defences in MASLD pathogenesis, further investigation is necessary to establish targeted interventions.

#### 3.2.5. Dysbiosis of the Gut Microbiota

There is increasing evidence that gut microbiota contributes to liver fat accumulation and inflammation involved in liver fibrosis (Figure 3). The gut microbiota of patients with MASLD exhibits characteristic dysbiosis, defined as abnormal qualitative and quantitative changes and altered proliferation or reduction in specific microbial communities, such as reduced abundance of beneficial commensal gut bacteria (e.g., *Akkermansia muciniphila* and *Faecalibacterium prausnitzii*), together with an increased abundance of endotoxin-producing bacteria, including Enterobacter and Escherichia coli [78,79,80,81]. Further, short-chain fatty acid (SCFA)-producing bacteria, such as Prevotella-9 and Ruminococcus, are reduced in MASLD patients [82], and changes in the abundance of bile acid metabolizing bacteria result in an imbalance in the ratio of primary to secondary bile acids, and alteration of the farnesoid X receptor (FXR) signalling pathway in liver and ileum tissues [83]. The key role of microbiota in MASLD was further confirmed by a study showing that a thyroid hormone receptor β (THR- β) agonist alleviated MASLD by regulating sphingolipid metabolism by intestinal microbes, particularly by increasing the relative abundance of Bacteroides [84].

Dysbiosis results in impairing intestinal tight junctions, which results in increased intestinal permeability, allowing harmful bacteria and their metabolites to reach the liver, through the enterohepatic circulation [85]. Lipopolysaccharide (LPS), a potent endotoxin derived from gut bacteria, alters the intestinal barrier and maintains intestinal inflammation by activating toll like receptors (TLRs). TLRs are pattern recognition receptors that participate in host defence by recognizing pathogen-associated molecular patterns (PAMPs) and activating innate immunity [86]. Furthermore, it has been shown that hepatocytes also express the TLR4 protein on the cell surface and can directly respond to LPS promoting hepatic inflammation [87]. In a diet-induced mouse model of MASLD, the LPS/TLR4 axis regulates hepatic glutaminase 1 (GLS) expression and promotes hepatic ammonia accumulation, leading to MASH progression [88]. Finally, LPS also induce Kupffer cells polarization toward the M1 phenotype resulting in higher levels of cytokines such as TNF-α, and IL-1β, along with ROS. This polarization amplifies hepatic inflammation and oxidative stress, key players in MASLD progression [89,90] (Figure 3).

Altogether, these data underscore an important role of the gut–liver axis in the pathogenesis of MASLD, linking gut microbiota dysbiosis with LPS-mediated signalling and the induction of hepatic inflammation.

#### 3.2.6. Genetic and Epigenetic Factors

Both genetic and epigenetic factors play crucial roles in the development and progression of MASLD. Genetic variations, such as polymorphisms, confer inherent susceptibility by affecting key pathways in lipid metabolism and inflammation. Meanwhile, epigenetic modifications dynamically regulate gene expression in response to environmental and lifestyle influences, further shaping disease onset and severity.

Functional polymorphisms in key metabolic genes can disrupt hepatic lipid homeostasis and insulin signalling pathways, thereby conferring disease susceptibility (Figure 3). Genome-wide association studies (GWAS) identified five core genes that regulate hepatic lipid metabolism, inflammation, and fibrosis: Patatin-like phospholipase domain-containing protein 3 (PNPLA3), Transmembrane 6 superfamily member 2 (TM6SF2), Membrane-bound O-acyltransferase domain-containing 7 (MBOAT7), Glucokinase regulator (GCKR), and Hydroxysteroid 17-beta dehydrogenase 13 (HSD17B13) [91]. The PNPLA3 allele (rs738409[G], encoding I148M) variant is the strongest genetic risk factor affecting TG hydrolysis and VLDL secretion, leading to lipid accumulation [92,93,94,95]. This mutation disrupts retinol release in hepatic stellate cells and inhibits ATGL-mediated lipolysis promoting inflammation and fibrosis [96,97]. Similarly, the encoded G to A substitution of TM6SF2 from glutamate to lysine at position 167 of the rs58542926 SNP leads to loss of function of the hepatic VLDL secretory pathway, which induces elevation of hepatic triglyceride levels and thus increases susceptibility to liver injury [98,99], but reduces cardiovascular risk by decreasing circulating lipids [100,101]. MBOAT7 rs641738 downregulates phosphatidylinositol synthesis, driving pro-inflammatory lipid species [102,103], while GCKR variants (e.g., rs1260326) enhance glucokinase activity to promote lipogenesis [104]. In contrast, HSD17B13 truncating variants (e.g., rs72613567) protect against fibrosis and HCC by attenuating inflammation via retinol metabolism [105,106,107]. These findings highlight the critical role of gene-environment interplay in MASLD and emphasize the therapeutic potential of targeting pathways such as PNPLA3-mediated lipolysis, MBOAT7-driven inflammation and HSD17B13-related retinol metabolism, offering new avenues for the development of targeted treatments.

On the other hand, epigenetic modifications including DNA methylation, RNA chemical modifications, histone modifications and non-coding RNAs, have also emerged as key regulators in the development and progression of MASLD, influencing gene expression without altering the underlying DNA sequence. For example, Pirola et al. found hepatic hypermethylation and reduced transcriptional activity of mitochondrially encoded NADH dehydrogenase 6 (MT-ND6) that correlated with histological severity of MASLD determined by liver biopsy in patients [108].

RNA modifications may also play a significant role in MASLD development, as evidenced by the identification of 2128 differentially N4-acetylcytosine (ac4C) acetylated sites in MASLD models [109]. For example, ac4C modification of Sterol regulatory element binding transcription factor 1 (Srebf1) and SREBF chaperone (Scap) mRNAs enhances hepatic lipogenesis and increases lipid deposition in the liver [110]. On the other hand, RNA methylation modulates RNA stability affecting its translation. In this sense, methyltransferase-like 14 (METTL14) overexpression both in *db*/*db* mice and MASLD patients suppresses Sirtuin 1 (SIRT1) activity, exacerbating IR and increasing lipid accumulation [111].

Histone acetylation, another key epigenetic modification, has been increasingly recognized for its role in regulating gene expression and metabolic pathways implicated in MASLD. Major acetylation sites on histone H3 include K27, a prevalent post-translational modification [112] which emerges as a potential therapeutic target. Thus, in preclinical models, reduced H3K27 acetylation levels have been shown to ameliorate hepatic steatosis [113], whereas enhanced acetylation upregulates fatty acid transporters (CD36), promoting intracellular lipid accumulation [114,115].

Finally, non-coding RNAs (ncRNAs), including micro RNAs (miRNAs), circular RNAs (circRNAs) and long non-coding (lncRNAs), play an important role in the development of MASLD as regulators of several biological processes. In Table 2, we show the newly discovered ncRNAs in recent years and their potential involvement in hepatic lipid metabolism and MASLD development.

Epigenetic modifications are well recognized to be primarily influenced by environmental and lifestyle factors such as regular physical activity, stress management, and a balanced diet. Consequently, adopting healthier lifestyle habits may help prevent detrimental epigenetic alterations associated with the development of MASLD. Collectively, understanding the interplay between genetic and epigenetic factors may contribute to the future development of targeted prevention and treatment strategies for MASLD.

### 3.3. Association Between MASLD and Atherosclerosis (AS)

The main cause of mortality in patients with MASLD is cardiovascular disease (CVD), surpassing liver complication-related deaths. There is a strong association between MASLD and the risk of developing CVD [127]. Specifically, patients with MASLD show a high risk of mortality from atherosclerotic CVD, due to increased synthesis of VLDL, IR and inflammation and endothelial dysfunction [128] (Figure 3).

MASLD patients exhibit characteristic dyslipidaemia marked by hypertriglyceridemia with elevated VLDL and intermediate-density lipoprotein (IDL) levels, increased low-density lipoprotein (LDL) particles, and reduced high-density lipoprotein (HDL) levels. These lipid abnormalities synergistically promote vascular lipid deposition and accelerate plaque formation through multiple pathways [129]. For example, the elevated ApoB-100 lipoprotein levels enhance arterial retention of atherogenic lipoproteins, exacerbating atherogenesis [130]. TG-rich lipoproteins, such as VLDL and IDL, which contain apolipoprotein C3, promote CVD development by stimulating pro-inflammatory mediators within the vascular wall [131]. Notably, reduced levels of ApoA-I leading to low HDL levels impaired reverse cholesterol transport, further compromising vascular homeostasis [132,133].

MASLD induces systemic metabolic perturbations that could exacerbate the atherosclerotic process. Thus, increased levels of circulating NEFA and lipotoxic metabolites (e.g., ceramides) cause endothelial dysfunction, oxidative stress and inflammation. In the context of IR, there is a decrease in endothelial production of nitric oxide (NO), increased expression of adhesion molecules, proliferation of vascular smooth muscle cells (VSMC), and oxidative stress, further promoting endothelial dysfunction Moreover, MASLD patients showed reduced tissue plasminogen activator (tPA), and increased plasminogen activator inhibitor-1 (PAI-1) levels, which collectively impair fibrinolytic capacity and enhance thrombotic risk [134,135].

Emerging evidence also establishes gut microbiota as a critical mediator in MASLD-associated atherosclerosis. Increased intestinal permeability in MASLD facilitates LPS translocation into systemic circulation, stimulating human monocytes to activate TLR4-MyD88-NF-κB signalling to produce proinflammatory cytokines (IL-1β, IL-6, TNF-α) while disrupting insulin signalling [136,137,138,139,140,141]. Trimethylamine *N*-oxide (TMAO), a microbial metabolite derived from choline/L-carnitine metabolism, promotes atherogenesis through multiple mechanisms: upregulating macrophage scavenger receptors (SR-A/CD36); suppressing hepatic CYP7A1 to reduce bile acid synthesis and cholesterol clearance; and activating GPCR/NF-κB/MAPK pathways that induce endothelial inflammation [142,143,144,145]. Interestingly, patients with MASLD have a higher concentration of serum TMAO compared to healthy controls [146]. These microbial-metabolic interactions create a self-perpetuating cycle linking hepatic dysfunction with cardiovascular pathogenesis.

Collectively, these pathophysiological mechanisms suggest that MASLD represents an important and independent risk factor for the development of atherosclerosis. However, further research is required to establish a definitive causal relationship between MASLD and atherosclerotic disease.

## 4. The Importance of Sexual Dimorphism in MASLD

**Figure 4:**
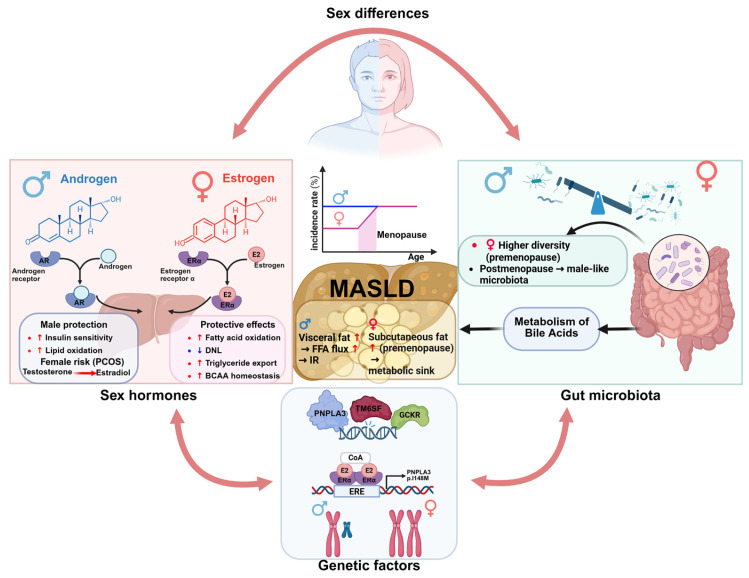
Mechanisms of Sexual Dimorphism in MASLD. Estrogen signalling through ERα suppresses lipogenesis and promotes fatty acid oxidation. The decline in estrogen post-menopause removes this protection, leading to a metabolic shift that predisposes to hepatic steatosis. This hormonal axis is modulated by sex-specific genetic factors and interacts bidirectionally with the gut microbiota, together defining the overall sexual dimorphism in MASLD presentation. Abbreviations: AR, androgen receptor; BCAAs, branched-chain amino acids; DNL, de novo lipogenesis; Erα, oestrogen receptor α; ERE, oestrogen response element; FFA, free fatty acid; GCKR, glucokinase regulator; IR, insulin resistance; MASLD, metabolic dysfunction-associated steatotic diver Disease; PCOS, polycystic ovary syndrome; PNPLA3, patatin-like phospholipase domain-containing protein 3; TM6SF2, transmembrane 6 superfamily member 2. This image was created with BioRender (https://biorender.com/).

Among adults in the general population, MASLD affects men more than women, with prevalence increasing during post-menopause. Sex differences are primarily attributed not only to sex hormones and related variations in fat distribution and IR, but also to lifestyle factors and environmental exposures not so directly related to hormonal differences. In addition, genetics and gut microbiome composition (Figure 4) have also been shown to impact MASLD sexual dimorphism.

### 4.1. Fat Distribution Patterns, IR and Environmental Factors

Distinguishing between sex and gender is crucial, as they are terms often treated as equivalent. Gender disparities involve societal, cultural, and lifestyle factors like diet, physical activity, and daily habits, and these factors also influence MASLD initiation and evolution. Women generally adopt healthier dietary practices than men, who tend toward riskier behaviours like tobacco use and alcohol consumption, although men typically engage in greater physical activity. However, these lifestyle differences do not primarily explain the variations in body composition between men and women. Men tend to accumulate visceral adipose tissue within the abdominal cavity, which is more sensitive to lipolysis and promotes hepatic steatosis via increased NEFA flux to the liver. Moreover, male fat expands through hypertrophy (enlargement of existing adipocytes), which are prone to inflammation and IR. In contrast, premenopausal women typically store more fat around their hips and thighs (subcutaneous fat). This gynoid pattern of adiposity functions as a protective metabolic sink, efficiently sequestering fatty acids and preventing them from spilling over into the liver. By safely storing these lipids, women avoid the formation of toxic lipid intermediates that disrupt insulin signalling and promote MASLD. Furthermore, female adipose tissue tends to expand through hyperplasia (the generation of new, smaller, and more insulin-sensitive adipocytes) which is strongly associated with maintaining a lower metabolic risk. However, menopause causes a shift toward central adiposity as estrogen levels decline, which explains, at least in part, the increased prevalence of MASLD in postmenopausal women [147,148].

### 4.2. Sex Hormones

The differences between men and women on body fat distribution and insulin sensitivity are heavily dependent on biological mechanisms, including hormonal regulation. Thus, MASLD sexual dimorphism is due, at least in part, to the estrogen’s protective role. Estrogen suppresses hepatic DNL, enhances fatty acid oxidation, and promotes TG export, reducing hepatic lipid accumulation [147]. Mechanistically, oestrogen receptor α (ERα) is pivotal in mediating sex-specific hepatic adaptations. ERα activation is responsible for the increase in lipid oxidation and DNL suppression, and maintains amino acid homeostasis, particularly branched-chain amino acids (BCAAs), which counteract diet-induced steatosis [149,150]. Accordingly, loss of ERα signalling, as occurs in postmenopausal women or ovariectomized mice, disrupts lipid metabolism, promotes toxic lipid accumulation, and accelerates MASLD progression [151,152]. Postmenopausal oestrogen decline heightens MASLD incidence and fibrosis risk, paralleling rates in age-matched males. Thus, clinical data reveal that MASLD prevalence in males peak at 50–60 years, while females show an upward trend post-menopause, reaching a zenith at 60–69 years [153]. However, clinical and mechanistic data reveal that the protective effects of oestrogens are neither absolute nor universal, and MASLD does occur in premenopausal women, particularly when metabolic risk factors are present.

Androgens also play a role in MASLD, exhibiting sexually dimorphic effects. In males, physiological testosterone levels enhance insulin sensitivity and lipid oxidation, protecting against hepatic steatosis [154]. However, hypoandrogenism in males and hyperandrogenism in females, such as in polycystic ovary syndrome (PCOS), exacerbate metabolic dysfunction. PCOS is associated with a 15–55% MASLD prevalence, with hyperandrogenaemia driving IR, visceral adiposity, and hepatic lipid accumulation [155]. Androgen excess in females upregulates lipogenic genes (e.g., SREBP-1, FASN) and pro-inflammatory pathways (e.g., NF-κB, TNFα) promoting MASH [156,157,158].

### 4.3. Sex-Related Genetic Factors

MASLD exhibits sex-specific genetic susceptibility, driven by polymorphisms (e.g., PNPLA3, TM6SF2, GCKR) with divergent effects across sexes [159]. Previous studies showed a strong association among the PNPLA3 rs738409 variant and increased ALT levels in men, regardless of age, and in women over 50 years [160,161]. Moreover, the recent work of Cherubini et al. suggests that the impact of PNPLA3 variants appears to be more pronounced in women, particularly postmenopausal, highlighting interactions between genetic risk and hormonal status [162]. Sex chromosomes also play a direct role in modulating the risk of metabolic disorders [147]. In humans, X chromosome abnormalities, such as Turner syndrome (TS), characterized by abnormal or absent X chromosome in females, further illustrate this [163]. Histological evidence of nodular hyperplasia, MASLD and cirrhosis have been described in women with TS. Accordingly, elevated liver enzymes were found in ∼50% of women with TS [164]. However, clinical data suggest that the adverse metabolic phenotype associated with TS (visceral obesity, IR, and dyslipidaemia) largely mediates the increased MASLD risk rather than sex chromosome loss alone.

### 4.4. Sex Differences in Gut Microbiota

As detailed in Section 3.2.5, the gut–liver axis plays a pivotal role in MASLD pathogenesis, so sex-specific differences in gut microbiota composition and metabolite signalling may contribute to distinct MASLD phenotypes between sexes. Overall, human studies show that gut microbiota composition differs between sexes, with females exhibiting higher diversity than males [165]. Notably, postmenopausal women harbour a gut microbiota composition more similar to that of men than that of premenopausal women [166]. Despite these differences, only a few studies have examined sex-related variations in gut microbiota between MASLD patients and healthy controls. A study in Chinese adults showed that men with MASLD exhibit reduced microbial diversity compared to healthy controls, whereas in women, MASLD was associated with increased diversity [80]. In contrast, another study in European population recently found that microbial diversity is primarily driven by MASLD status independently of sex, although specific microbial taxa changes show sex-specific patterns [167]. Sex hormones and gut microbiota engage in bidirectional crosstalk. Thus, FXR, a key bile acid sensor, interacts with estrogen to regulate microbial composition, while gut microbiota, in turn, influence sex hormone levels and hepatic sexual dimorphism [168,169]. These studies might uncover novel sex-specific microbiota-derived metabolites as MASLD biomarkers.

In summary, sex hormones play an important role shaping fat distribution and insulin sensitivity, interacting with genetic variants and gut microbiota to drive MASLD sexual dimorphism. Although clinical trials often overlook sex differences, evidence suggests that considering reproductive stage, hormone levels, and genetic variants can improve risk stratification and treatment outcomes [20]. Additionally, lifestyle interventions such as diet and exercise may yield different benefits by sex. For example, aerobic activity produces the greatest benefits in terms of improved liver markers in women, while men benefit more from anaerobic exercise [170]. Overall, integrating sex-specific biological and gender-related factors into MASLD management may support the development of more effective precision medicine strategies tailored to individual patient profiles.

## 5. Dietary-Induced MASLD Animal Models

Animal models that accurately reflect the pathology are vital to understand MASLD biology and to assess potential drug candidates. Although there are experimental models based on genetic modifications, they usually display only a few characteristics of the human disease. In contrast, as diet-induced obesity is the major driving force for MASLD in humans, dietary models better recapitulate the origin of the human disease. Although there is a wealth of experimental data about MASLD generated in mice models, rats are somehow more adequate than mice for diet-driven MASLD models, because it has been reported that they are more susceptible to high-fat diets (HFD) and show more severe and/or earlier histological features of MASLD characteristics, including fibrosis, especially the Sprague–Dawley strain [171,172]. Moreover, rats differ from mice in their inflammatory responses and cholesterol metabolism, being closer to humans in these aspects [173]. Our research group has been investigating liver metabolic alterations of dietary origin (fructose and fat) in rat models, characterizing a model of simple hepatic steatosis in Sprague–Dawley female rats and studying the anti-steatotic effect of natural products and drugs (bempedoic acid, telmisartan, pemafibrate) in this model [174,175,176]. For these reasons, this section focuses on MASLD dietary models established in rats, summarizing various dietary components and their impacts on histological outcomes and metabolic disturbances (Table 3).

### 5.1. High-Fat Diet

HFD models are extensively applied in rats to investigate MASLD, particularly when the research focus is the metabolic syndrome axis, including obesity, IR, dyslipidaemia, and hepatic steatosis. In rat studies, HFD typically provides ~45–75% of total caloric intake from fat, with saturated fatty acids being common drivers of lipid accumulation, glucose intolerance, and hyperlipidaemia [177,178,179]. Importantly, the hepatic phenotype in rats is highly contingent on strain, sex, dietary lipid composition, and the feeding duration; thus, “HFD” should be specified by fat source, fatty-acid profile, and *n*-6:*n*-3 ratio, rather than by fat percentage alone [195,196]. Among commonly used rat strains, Sprague–Dawley (SD) rats presented macrovesicular steatosis accompanied by pronounced fibrosis when fed an HFD [180]. In SD rats, a HFD containing 60% fat and 20% carbohydrates induces steatosis and inflammation as early as 16 weeks [181]. Prolonged HFD feeding for 43 or 48 weeks results in hepatic lobular inflammation and perisinusoidal fibrosis, which become evident after 36 weeks, making this model particularly valuable for advanced MASLD studies [182,183]. Collectively, these observations support HFD-fed rats as a suitable platform for studying the transition from metabolic stress to hepatic injury, especially when time-dependent progression is a core objective.

Beyond macronutrient ratios, the type, chain length, conformation and *n*-6:*n*-3 ratio of dietary fatty acids have a key influence on the development and progression of MASLD. Long-chain fatty acids (LCFA) promote impaired glucose tolerance and accelerated fibrosis more than diets containing medium-chain fatty acids (MCFA), such as those found coconut oil [197]. Western diets rich in *n*-6 PUFA induce steatosis, oxidative stress, and fibrosis in rodents [198]; meanwhile, excessive intake of trans fatty acids is closely associated with liver fibrosis, and partial PUFA replacement attenuates LPS-induced inflammatory responses [199]. These findings provide an experimental basis for the development of rational dietary intervention strategies, suited for metabolic endpoints (body weight/adiposity, IR, dyslipidaemia), early intermediate MASLD mechanisms, and drug testing where metabolic improvement is a key outcome. However, this model is less suited for rapid generation of advanced fibrosis; severe inflammatory/fibrotic endpoints may require very long duration, specific lipid composition, and/or combination with other dietary hits.

### 5.2. High-Fat and High-Cholesterol Diet (HFHCD)

Cholesterol-enriched diets were initially utilized in atherosclerosis research, and subsequent studies have demonstrated their synergistic effects with HFD (HFHCD), significantly promoting hepatic lipid accumulation [184]. An atherogenic dietary regimen comprising 10% lard and 2% cholesterol (*w*/*w*) has been shown to progressively induce hepatic steatosis, necrosis, ballooning, steatohepatitis, and fibrosis, alongside metabolic abnormalities such as obesity, dyslipidaemia, IR, and elevated serum aminotransferase levels in rats [183]. Thus, relative to HFD alone, HFHCD generally provides a stronger platform for studies that require concurrent assessment of metabolic endpoints and hepatic inflammatory/fibrotic progression. Recent animal models further confirm that SD rats consuming an HFHCD supplemented with bile acids exhibit an accelerated progression of MASH-like lesions, highlighting the critical role of enhanced intestinal cholesterol absorption in disease pathogenesis [185]. Thus, cholic acid (CA) supplementation under high-fat conditions exacerbates liver inflammation and fibrosis in cholecystectomized SD rats, leading to the establishment of a novel MASH model [200]. In this context, HFHCD-based protocols may also be useful for investigating the gut–liver axis, bile acid signalling, and cholesterol handling, provided that experimental design explicitly disentangles lipid overload from bile acid-driven effects.

While HFHCD improves face validity for “Westernized” dietary exposures, it remains important to emphasize that rats rarely reproduce the entire human MASLD/MASH disease spectrum (particularly late-stage inflammatory complications and hepatocellular carcinoma) without additional modifiers or long-term interventions. Moreover, rat cholesterol handling and immune-inflammatory responses are not exactly as in humans, which can influence both disease kinetics and therapeutic response, especially in cholesterol-driven injury settings. Therefore, HFHCD is best interpreted as a model to interrogate diet-driven metabolic hepatic injury coupling rather than a full replica of natural disease history.

### 5.3. High-Fructose Diet

As a commonly used sweetening additive, fructose not only induces DNL and IR, but also inhibits fatty acid oxidation and triggers endoplasmic reticulum stress. In recent years, many animal models of MASLD have incorporated fructose supplementation in liquid or solid form, either alone or in combination with high-fat and cholesterol diets to more realistically recapitulate the mechanisms of the disease in humans [186]. In rats, fructose-based paradigms are particularly informative when the study endpoint includes hepatic DNL, lipid partitioning, insulin signalling, and diet–microbiome interactions, while the magnitude and direction of metabolic outcomes may be sex and time dependent.

Previous studies from our research group have demonstrated that supplementing male rats fed a standard diet with 10% fructose in drinking water for 14 days induced hypertriglyceridemia and hepatic steatosis, primarily through two mechanisms: increased lipogenesis and decreased hepatic β-oxidation [201,202]. Similar experiments in female rats produced comparable results, but unlike males, female rats developed IR [203]. Furthermore, in females, fructose supplementation altered the endoplasmic reticulum stress pathway. Longer fructose exposure in female rats (8 weeks) caused hyperinsulinemia and a pronounced reduction in insulin receptor substrate 2 (IRS-2), a key mediator of hepatic insulin signalling, yet their insulin sensitivity index was unaltered, suggesting that the elevated insulin levels were sufficient to maintain normal plasma glucose concentration [204]. The metabolic alterations induced by fructose are dynamic and depend on several factors, including the duration of supplementation. Thus, our group has shown that some effects are evident after short-term exposure but may diminish with prolonged treatment [205,206,207]. These findings underscore that in rat fructose models, timing of phenotyping (early vs. late) and sex stratification are essential to avoid misclassification of IR status or hepatic insulin signalling changes.

Recently, our group characterized a model of the first stage of MASLD, simple hepatic steatosis, by administering female rats a high-fat diet devoid of cholesterol supplemented with 10% *w*/*v* fructose in drinking water for three months (HFHFr) [175]. This diet induced hepatic steatosis in the absence of other metabolic disorders (e.g., obesity, inflammation, or type 2 diabetes mellitus) and induced changes in the fecal microbiota of rats [176,188,189]. In contrast, males exposed to the same dietary challenge exhibit hypertriglyceridemia, but not hepatic steatosis, partially due to enhanced liver β-oxidation and to their ability to expand the adipose tissue [189]. Collectively, these data support the use of rats supplemented with fructose (alone or combined) as a flexible platform for dissecting sex-specific metabolic partitioning (hepatic vs. adipose), with endpoints extending from steatosis initiation to microbiota-associated changes.

### 5.4. Methionine and Choline Deficient Diet (MCD)/Choline-Deficient, L-Amino-Defined Diet (CDAA)/Choline-Deficient Diet (CDD)

MCD, CDAA, and CDD induce MASLD and fibrosis through similar nutrient deprivation mechanisms. The MCD impairs VLDL secretion and mitochondrial function, triggering rapid hepatic steatosis (2–4 weeks), necroinflammation, and fibrosis, albeit with paradoxical weight loss and absent IR, key discordances from human MASLD phenotypes [190,191]. In rats, this diet predominantly causes severe hepatic steatosis with attenuated inflammatory/fibrotic responses [208]. CDAA diets also cause hepatic steatosis and fibrosis by the same mechanism, but in contrast to MCD, it partially alleviates weight loss, although it fails to replicate metabolic syndrome components, primarily modelling fibrosis and progression to hepatocarcinogenesis [192,193]. CDD diets provoke milder steatosis with transient metabolic dysregulation [194]. While all three models recapitulate histopathological MASH hallmarks (ballooning degeneration, collagen deposition), their translational use is limited by the absence of obesity/hyperlipidaemia and species-specific differences (mouse: inflammation-fibrosis vs. rat: steatosis predominant) factors, which make them primarily useful for histopathological, rather than metabolic studies.

## 6. Latest Advances in MASLD Therapy

In the evolving landscape of drug development for MASLD/MASH, the limitations of monotherapy have become increasingly apparent. Several key therapeutic targets such as thyroid hormone receptor-β (THR-β), glucagon-like peptide-1 (GLP-1) receptor, fibroblast growth factors (FGFs), FXR, free fatty acid receptors (FFARs), and PPARs have been actively explored [209,210]. However, in clinical studies, monotherapies have demonstrated a high failure rate or an inability to meet primary endpoints. As stated in the introduction section, nowadays there are only two drugs approved specifically for the treatment of non-cirrhotic MASH with moderate-to-advanced fibrosis (stages F2–F3), resmetirom and semaglutide.

**Figure 5:**
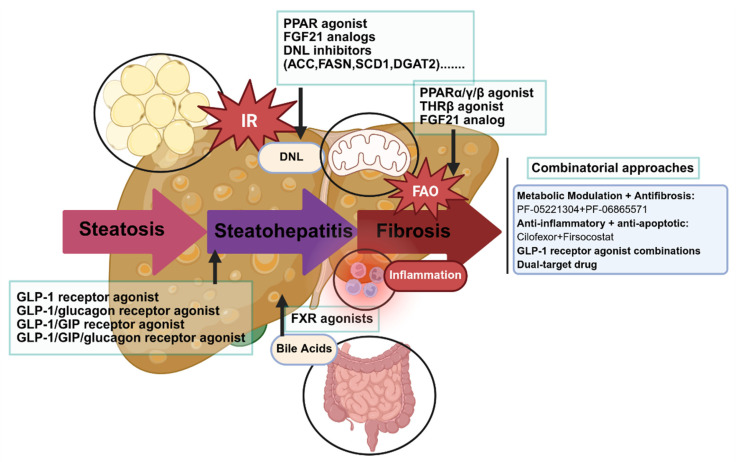
Pharmacological targets of MASLD therapy. By simultaneously modulating key pathways involved in metabolism (through modulation of THR-β, GLP-1and FGFs), inflammation, and fibrosis (through FXR agonists and CCR2/5 antagonists), these strategies aim to create a synergistic therapeutic effect that surpasses the efficacy of any single agent, thereby addressing the multifaceted nature of the disease. Abbreviations: ACC, acetyl-coA carboxylase; CCR2/5: CC-chemokine receptor 2/5; DGAT2, diacylglycerol O-acyltransferase 2; DNL, de novo lipogenesis; FAO, fatty acid oxidation; FASN, fatty acid synthase; FGF21, fibroblast growth factor 21; FXR, farnesoid X receptor; GIP, glucose-dependent insulinotropic polypeptide; GLP-1, glucagon-like peptide-1; IR, insulin resistance; PPAR, peroxisome proliferator–activated receptors; SCD1, stearoyl-coA desaturase 1; THR, thyroid hormone receptor. This image was created with BioRender (https://biorender.com/).

In MASLD, a multisystem disease involving complex and intertwined signalling cascades, targeting individual pathways may result in limited or even conflicting therapeutic effects. An emerging strategy aims to modulate multiple disease pathways simultaneously, addressing hepatic steatosis, inflammation, and fibrosis through complementary mechanisms. Thus, some phase 2 trials have evaluated combinations of agents targeting different mechanisms (Figure 5). For instance, the ATLAS trial assessed the efficacy of the FXR agonist cilofexor, the ACC inhibitor firsocostat, and the anti-apoptotic agent selonsertib, alone or in pairs, in patients with MASH. While the combination of cilofexor and firsocostat showed synergistic effects in improving hepatic steatosis and biochemical markers, the trial failed to meet its primary endpoint of ≥1-stage fibrosis improvement without worsening of MASH [211].

The GLP-1 receptor agonist semaglutide, when combined with firsocostat and/or cilofexor, led to greater improvements in hepatic steatosis and serum liver enzymes than semaglutide alone in patients with MASH and mild to moderate fibrosis [212]. In another study, combining a GLP-1 receptor agonist with efruxifermin, a fibroblast growth factor 21 (FGF21) analogue, showed significant reductions in liver fat content, fibrosis markers, and metabolic parameters in patients with MASH and type 2 diabetes [213]. Other combination strategies aim to integrate systemic metabolic regulation with localized anti-inflammatory effects. For example, tropifexor a selective, non-bile-acid FXR agonist is being tested in combination with agents such as licogliflozin (a sodium glucose cotransporter inhibitor) or cenicriviroc, a dual chemokine receptor type 2/5 (CCR2/CCR5) antagonist in several phase 2 trials to evaluate safety and potential efficacy [214].

Drug combination can also be used to reduce side effects of the therapy. For example, one study combined the ACC inhibitor PF-05221304 with the diacylglycerol O-acyltransferase 2 (DGAT2) inhibitor PF-06865571. This combination significantly reduced hepatic fat content in patients with MASLD while mitigating the hypertriglyceridemia typically associated with ACC inhibition, illustrating the complementary potential of mechanism-based combinations [215].

In addition to combination therapy, novel drug candidates with dual-target mechanisms are emerging. In a study with survodutide, a dual GLP-1/glucagon receptor agonist, 83% of participants achieved improvement in MASH with no worsening in fibrosis, and 64% of participants with F2–3 fibrosis had ≥1 stage improvement of fibrosis without worsening of MASH [216]. Another dual glucose-dependent insulinotropic polypeptide (GIP)/GLP-1 receptor dual agonist tirzepatide, showed significant histological improvement in 62% of patients receiving the 15 mg dose, with fibrosis improvement also significantly higher than placebo [217].

However, although all the above-mentioned phase 2 studies showed that the drugs involved were mostly well tolerated, with a low incidence of mild adverse effects (pruritus, gastrointestinal symptoms, etc.), they were small-scale trials including a small number of patients. Double-blind placebo-controlled phase 3 trials with adequate patient numbers are warranted to properly assess the efficacy and safety of these drug combinations in MASH.

The increasing accumulation of drug–target–disease information, combined with the refinement of predictive models thanks to data mining and artificial intelligence, makes drug repurposing an attractive approach, potentially enabling the discovery of new indications for existing drugs beyond their initial medical purpose. Specifically, the repositioning of drugs that have demonstrated to be safe and have completed phase I clinical studies can increase the approval success rate up to 30%. As the complexity of MASLD increases exponentially with the temporal evolution of the pathology, to investigate drug repurposing strategies for MASLD, our group employed a female SD rat model of simple hepatic steatosis induced by a HFHFr diet. Using this model, we observed that bempedoic acid (BemA), an ACLY inhibitor used as a hypolipidemic drug, exhibits potent anti-steatotic effects. These effects are mediated by the activation of PPARα, enhancement of hepatic fatty acid β-oxidation, upregulation of PNPLA3 (thereby promoting triglyceride efflux), and inhibition of fructose uptake and lipid synthesis via KHK inhibition [176]. Furthermore, BemA restores hepatic H_2_S production by modulating the FXR/PGC1α pathway, effectively counteracting the H_2_S deficiency observed in liver disease [218]. Similarly, pemafibrate, a selective PPARα modulator licenced in Japan to treat dyslipidaemia, almost completely reversed hepatic steatosis in this model, primarily through the potent activation of PPARα-driven fatty acid catabolism. Recent findings indicate that pemafibrate also significantly alters fecal bile acid composition and gut microbiota structure [188]. The angiotensin II receptor (AGTR) antagonist telmisartan, widely used for the treatment of hypertension, also exhibited pronounced anti-steatotic effects. Our group’s findings indicate that its mechanism is PPAR-independent; instead, it redirects fructose metabolism from lipogenesis towards gluconeogenesis and the polyol pathway by upregulating hepatic PCK1 expression. Moreover, AGTR1 blockade also contributes to this effect [219]. In contrast, the β3-adrenergic receptor agonist mirabegron, clinically used to treat urinary incontinence, failed to reduce hepatic lipid accumulation in this model, despite increasing UCP1 expression in brown adipose tissue and β3-adrenergic receptor expression in relevant tissues. Intriguingly, mirabegron was found to alter the gut microbiota composition; however, this effect was independent of bile acids and likely represents a direct pharmacological action [220]. Collectively, these findings from a specific preclinical model not only provide support for the potential repurposing of drugs such as bempedoic acid, pemafibrate and telmisartan for MASLD treatment, but also reveal novel mechanisms and pathways (e.g., H_2_S and PCK1), thereby deepening our understanding of MASLD pathophysiology.

## 7. Future Research Directions

Despite significant progress in MASLD research, numerous challenges persist, owing to its complex and heterogeneous nature. Early intervention in MASLD represents a critical opportunity to alter disease trajectory before irreversible damage occurs. In its initial stages, MASLD is largely driven by metabolic dysfunction and simple steatosis, a condition traditionally regarded as highly reversible. Targeting the disease at this point, before inflammation and fibrogenesis become established, offers the possibility of restoring metabolic homeostasis, but the benign nature of simple steatosis requires agents with a favourable safety profile, such as repurposed cardiovascular drugs. Future investigations should also focus on mechanisms directly linked to disease progression, including how ERα and androgen imbalance influence the transition from steatosis to MASH and fibrosis. Clarifying how the gut microbiota and its metabolites (e.g., TMAO and SCFAs) modulate the gut–liver axis through dynamic epigenetic modifications may also help identify patients at risk of fibrotic progression. Furthermore, exploring the interplay among ferroptosis, lipotoxicity, and oxidative stress may facilitate the development of novel interventions that target hepatocellular injury.

Given the inherent challenges in extrapolating findings from preclinical models to humans, future efforts should focus on developing animal models that better capture the metabolic diversity, genetic backgrounds, and environmental influences observed in MASLD patients. Such improvements are essential for validating pathogenic mechanisms and predicting therapeutic responses in a clinically meaningful way. At the same time, the integration of multi-omics datasets offers a powerful opportunity to dissect this heterogeneity, but its success will depend on accounting for differences in diet, lifestyle, microbiome composition, and other environmental exposures.

Promising drug classes for MASLD may include those that address multiple pathogenic nodes simultaneously, such as dual GLP-1/GIP and GLP-1/glucagon receptor agonists. Progress in this field will also depend on improving non-invasive diagnostic tools and trial endpoints to accelerate drug development and better capture fibrotic progression. The recent FDA qualification (August 2025) of liver stiffness measurement by vibration-controlled transient elastography, as a reasonably likely surrogate endpoint for clinical trials in non-cirrhotic MASH with moderate-to-advanced fibrosis, represents a key step in this direction. Together, efforts to understand the biological processes that trigger disease development, optimize animal models, enhance non-invasive diagnostic techniques, and promote interdisciplinary research may pave the way toward more precise, effective, and patient-centred therapies for managing MASLD.

## Figures and Tables

**Table 2:** Examples of dysregulated non-coding RNA biomarkers in MASLD.

Type of ncRNA	Modification (Role)	Mechanism	References
miRNAs	miR-33 ↑	Overexpression of miR-33 inhibits the expression of genes involved in fatty acid oxidation and insulin signalling promoting MASLD progression in hepatic cells.	[116]
	miR-149-5p ↑	miR-149-5p upregulation significantly affects hepatocyte energy metabolism and exacerbates hepatic steatosis and inflammation/fibrosis by regulating an extensive network of target genes	[117]
	miR-411-5p ↓	Decreased hepatic miR-411-5p levels are associated with increased lipid deposition due to enhanced lipid synthesis, likely as a consequence of elevated EIF4G2 and FOXO3 expression levels.	[118]
	miR-34a-5p ↑	miR-34a-5p targets and downregulates SIRT1 expression, a multifunctional protein involved in regulating lipid synthesis, lipid oxidation, inflammation, oxidative stress, and other processes implicated in lipid deposition and MASLD development.	[119]
	miR-511-3p ↓	miR-511-3p targets and promotes the degradation of ROCK2 mRNA, thereby reducing its translation. Dysregulation of miR-511-3p levels in MASLD leads to increased ROCK2 expression, inflammation and liver fibrosis development	[120]
circRNAs	circRNA RCRIN ↓	Lower RCRIN levels lead to the release of RPL8 to form RPL8-containing ribosomes, promoting lipid accumulation and endoplasmic reticulum stress.	[121]
	mmu_circ_0009303 ↑	Mmu_circ_0009303 promotes oxidative stress, inflammation and excessive fat accumulation by regulating the miRNA-182-5p/Foxo3 axis and lipid metabolism-associated regulatory proteins	[122]
	circRRM2 ↓	CircRRM2 functions as a miR-142-5p sponge, reducing its availability. Consequently, decreased levels of CircRRM2 result in elevated miR-142-5p levels, which trigger the upregulation of lipogenesis-related genes and increased triglyceride accumulation in the livers of MASLD mice.	[123]
lncRNAs	TCONS-00039830 ↑	Upregulation of TCONS_00039830 increases SMAD2 expression and decreases miR-455-3p levels in the liver, thereby promoting fat accumulation.	[124]
	H19 ↑	lncRNA H19 expression augments DNL via PPARγ-mediated SREBP1c activation and inhibits fatty acid oxidation.	[125,126]
	NORAD ↑	NORAD directly binds to and stabilizes ROCK2 by reducing Nedd4-mediated ubiquitination of ROCK2, thereby affecting the MASLD process. The NORAD/ROCK2 activation axis has been shown to increase liver fibrosis and inflammation.	[120]

Arrow symbols indicate changes in non-coding RNA expression in MASLD: ↑ indicates upregulation, whereas ↓ indicates downregulation.

**Table 3:** Comparative Analysis of Dietary Models for MASLD Induction.

Diet Model (Rat)	Characteristics	Main Phenotypes (Hepatic/Metabolic)	Translational Relevance (Strengths)	Major Limitations	Best Fit Use	Key References
HFD	45–75% kcal fat	Liver: predominant steatosis; Metabolism: obesity, insulin resistance, dyslipidaemia	Closely mimics overnutrition-driven metabolic syndrome and early MASLD	Fibrosis may be inconsistent without long-term feeding; phenotype depends strongly on fat composition; rats rarely progress to full late-stage spectrum	Metabolic syndrome, IR, lipid metabolism; early fibrosis in long-term protocols	[177,178,179,180,181,182,183]
HFHCD	Non-physiological Cholesterol (1–2%) + cholic acid +High-fat	Liver: steatosis, ballooning, inflammation, fibrosis; Metabolism: obesity, insulin resistance, elevated ALT	Preserves metabolic abnormalities while more reliably promoting MASH-like pathology	Species differences in cholesterol handling and immune responses may affect external validity; still rarely reproduces full human end-stage spectrum	MASH pathology, anti-inflammatory/anti-fibrotic drug testing, extrahepatic cardiovascular outcomes	[184,185]
High fructose	10–20% *w*/*v* Fructose; often combined with HFDs (HFHFr)	Liver: steatosis (fructose alone); Metabolism: insulin resistance and dyslipidaemia (sex and time dependent)	Well suited for dissecting de novo lipogenesis, insulin signalling, and sex-specific metabolic responses	Alone may not robustly drive fibrosis; dynamic adaptation can obscure endpoints if timing is not controlled	DNL, IR, ER stress, sex differences; early MASLD	[176,186,187,188,189]
MCD	Methionine + Choline deficient	Liver: severe steatosis; Metabolism: weight loss, absence of insulin resistance	Rapid and reproducible induction of hepatic histopathological injury	Weight loss and absent IR discordant from human MASLD; rat phenotype tends to be steatosis-predominant	Histopathology focused studies, oxidative stress, fibrogenic mechanisms	[190,191]
CDAA	Choline deficient + L-amino acid defined	Liver: steatosis → fibrosis → cirrhosis/HCC; Metabolism: no obesity or IR	Enables investigation of fibrosis to carcinogenesis continuum	Deficiency-driven and not representative of overnutrition; rat responses may diverge from human immunometabolic context	Fibrosis progression, hepatocarcinogenesis related mechanisms	[192,193]
CDD	Choline deficient	Liver: mild to moderate steatosis with limited fibrosis	Mild and technically simple model	Weak inflammatory/fibrotic responses; poor metabolic relevance	Mild steatosis, choline-metabolism related studies	[194]

## Data Availability

No new data were created or analyzed in this study. Data sharing is not applicable to this article.
